# Shrinkable silver diffraction grating fabricated inside a hydrogel using 522-nm femtosecond laser

**DOI:** 10.1038/s41598-017-17636-z

**Published:** 2018-01-09

**Authors:** Manan Machida, Yasutaka Nakajima, Maria Leilani Torres-Mapa, Dag Heinemann, Alexander Heisterkamp, Mitsuhiro Terakawa

**Affiliations:** 1 School of Integrated Design Engineering, Keio University, 3-14-1, Hiyoshi, Kohoku-ku, Yokohama 223-8522 Japan; 2 Institut für Quantenoptik, Gottfried Wilhelm Leibniz Universität Hannover, Am Welfengarten 1, 30167 Hannover, Germany; 3 Industrial and Biomedical Optics Department, Laser Zentrum Hannover e.V., Hollerithallee 8, D-30419 Hannover, Germany; 4 Department of Electronics and Electrical Engineering, Keio University, 3-14-1, Hiyoshi, Kohoku-ku, Yokohama 223-8522 Japan

## Abstract

The integration of metal microstructures and soft materials is promising for the realization of novel optical and biomedical devices owing to the flexibility and biocompatibility of the latter. Nevertheless, the fabrication of three-dimensional metal structures within a soft material is still challenging. In this study, we demonstrate the fabrication of a silver diffraction grating inside a biocompatible poly(ethylene glycol) diacrylate (PEGDA) hydrogel by using a 522-nm femtosecond laser *via* multi-photon photoreduction of silver ions. The optical diffraction pattern obtained with the grating showed equally spaced diffraction spots, which indicated that a regular, periodic silver grating was formed. Notably, the distance between the diffraction spots changed when the water content in the hydrogel was reduced. The grating period decreased when the hydrogel shrank owing to the loss of water, but the straight shapes of the line structures were preserved, which demonstrated the optical tunability of the fabricated structure. Our results demonstrate the potential of the femtosecond laser-based photoreduction technique for the fabrication of novel tunable optical devices as well as highly precise structures.

## Introduction

Hydrogels are promising flexible and elastic materials for biomedical applications, including tissue scaffolding^1,2^, drug delivery^3,4^, and biosensing^5^, owing to their high water retention capacity and biocompatibility. Because many hydrogels have high optical transmittance in the visible and near-infrared wavelengths, they have been considered for several emerging optical applications such as hydrogel-based light waveguide for optogenetics^6^ and hydrogel-based grating for optical sensing^7–9^ in recent years. The scope of use of hydrogels is expanding further because of their integration with metal micro- or nanostructures, which has increased the interest of researchers in the realization of novel electrical, optical, and micro-mechanical devices. There have been previous studies in which electrical conductivity^10^, bending capability^11^, and variable optical property^12^ were incorporated into hydrogels by integrating with metal structures. In a study by Shimamoto *et al*.^12^, a gold nano-dot array fabricated on the surface of a hydrogel showed variable resonant wavelengths depending on the extent of shrinking and swelling of the hydrogel. Although the fabrication of precise and functional metal structures was demonstrated using two-dimensional (2D) methods such as photolithography and transfer printing, the realization of three-dimensional (3D) structures is still a challenging task.

A femtosecond laser enables the fabrication of arbitrary 3D metal microstructures by multi-photon photoreduction of metal ions tightly confined to the focal point of the laser pulses. Many papers have reported the fabrication of metal structures based on multi-photon photoreduction^13–20^. The fabrication of a 3D self-standing silver gate microstructure on a glass substrate was demonstrated by Tanaka *et al*.^21^. The electrical^22–24^ and optical^25^ properties of the fabricated structures have been evaluated. It is acknowledged that the multi-photon photoreduction technique is applicable to the fabrication of metal microstructures not only in a liquid, but also within a supporting base material^26–33^. However, the fabrication of metal structures inside soft materials has been reported only during the last few years^34,35^.

In this paper, we report the fabrication of silver line structures inside a shrinkable hydrogel by multi-photon photoreduction of silver ions using a 522-nm femtosecond laser. The silver grating, which has homogeneous widths of the line structures, was fabricated inside a hydrogel to obtain an optical diffraction pattern. We have attempted to demonstrate the optical tunability of the fabricated silver grating by taking advantage of the shrinking property of the hydrogel.

## Results and Discussion

### Fabrication of silver line structures inside the hydrogel

**Figure 1:**
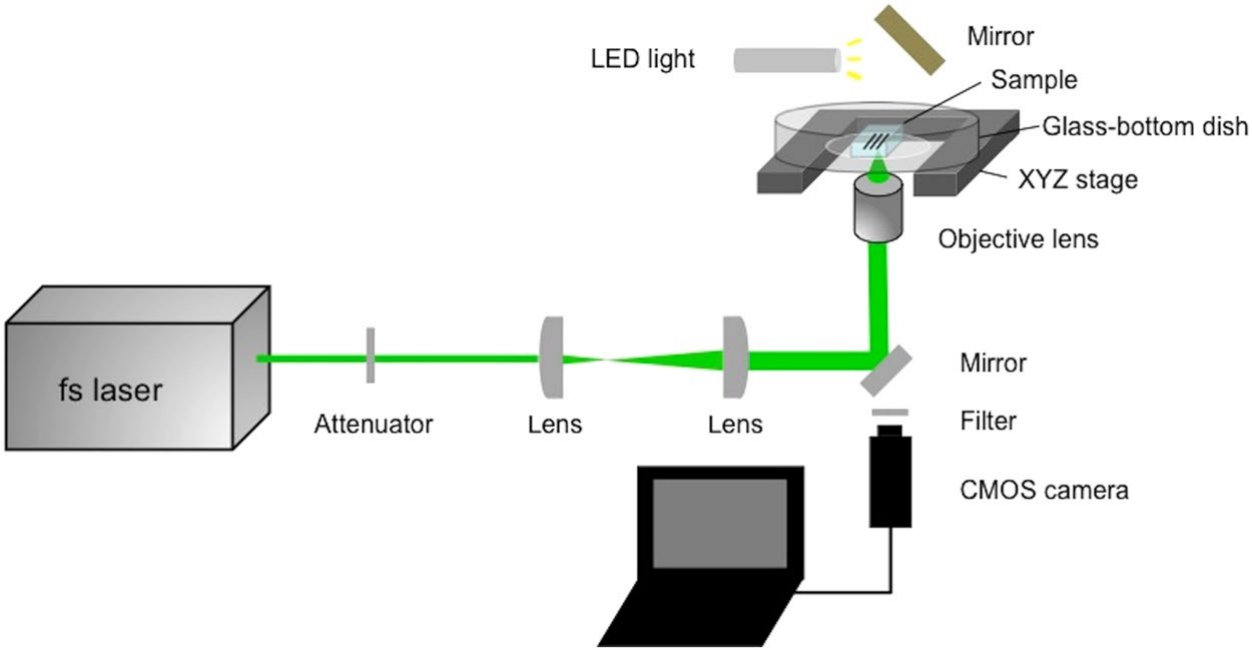
Schematic illustration of the experimental setup used for fabrication of silver line structures inside a hydrogel using a 522-nm femtosecond laser.

**Figure 2:**
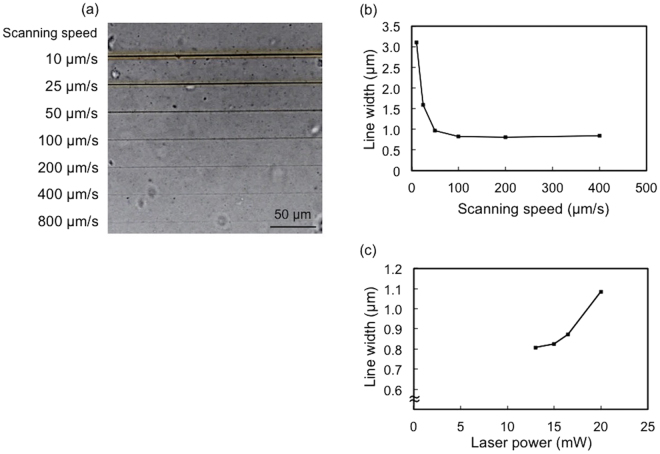
Silver line structures fabricated inside the hydrogel under different experimental conditions: (**a**) Bright-field microscope image of silver line structures fabricated with different scanning speeds of up to 800 μm/s. The laser power was 16.5 mW. A 10× (NA: 0.45) magnification microscope objective was used for obtaining the image. (**b**) Dependence of line widths of the silver microstructures on the scanning speed derived from the gray values of the microscope images obtained with a 60× (NA: 0.95) magnification microscope objective. The laser power was 16.5 mW. (**c**) Dependence of the line widths of the silver microstructures on laser power derived from gray values of the microscope images obtained with a 60× (NA: 0.95) magnification microscope objective. The scanning speed was 200 μm/s.

Figure 1 shows the experimental setup for the fabrication of silver line structures inside the hydrogel by multi-photon photoreduction of silver ions. A femtosecond laser oscillator that delivers laser pulses of 522-nm wavelength was used for the fabrication. Figure 2a shows the bright-field microscope image of the silver line structures fabricated inside a hydrogel at different scanning speeds by using a three-axis encoded (XYZ) motorized stage. The laser power was 16.5 mW (corresponding to energy of 0.26 nJ per pulse). Structures with homogeneous lines of different widths were fabricated at each scanning speed. Figure 2b shows the dependence of line width on the laser scanning speed. The full width at half maximum (FWHM) was derived from the gray values of the bright-field microscope images. The line width of the silver line structure fabricated with a scanning speed of 10 μm/s was ~3.1 μm. The line width was larger than the size of the theoretical diffraction limit of the laser beam at the focal point. This might be explained by the growth of metal particles^17,18^ or, possibly, by heat accumulation as well as the plasmon enhancement of optical intensity on the fabricated silver line structure by the nano-sized sub-structures resulting from multiple pulse overlaps. The line width of the fabricated structures remained constant (approx. 0.8 μm) at all scanning speeds higher than 100 μm/s. This could be due to a limitation in the optical resolution of the microscope; however, it should be noted that the gray values of the structures decreased with increasing scanning speed, as shown in Fig. 2a. The line widths fabricated at the scanning speeds higher than 800 μm/s were difficult to measure due to the low contrast of the gray value. These results suggest that the density of photo-reduced silver decreases with increasing laser scanning speed. The result is better understood by the optical intensity distribution of the laser beam and the threshold of optical intensity for multi-photon photoreduction. Because a high-repetition rate (63 MHz) laser oscillator was used in this study, the area subjected to irradiation by laser intensity exceeding the threshold of multi-photon photoreduction could be assumed to be a continuous scanned line at the highest scanning speed used in the study (800 μm/s). A decrease in the pulse overlaps, i.e., a decrease in the number of opportunities for photoreduction, could lower the density of silver formed in the scanned area. Figure 2c shows the dependence of line widths of the structures on the laser power. The line widths of the structures increased with increasing laser power. This is attributed to the Gaussian profile of the laser beam. The area subjected to the laser intensity exceeding the threshold of multi-photon photoreduction increased with increasing laser power. The structures fabricated with laser power higher than 22.0 mW were inhomogeneous (data not shown) and, therefore, line widths could not be determined.

**Figure 3:**
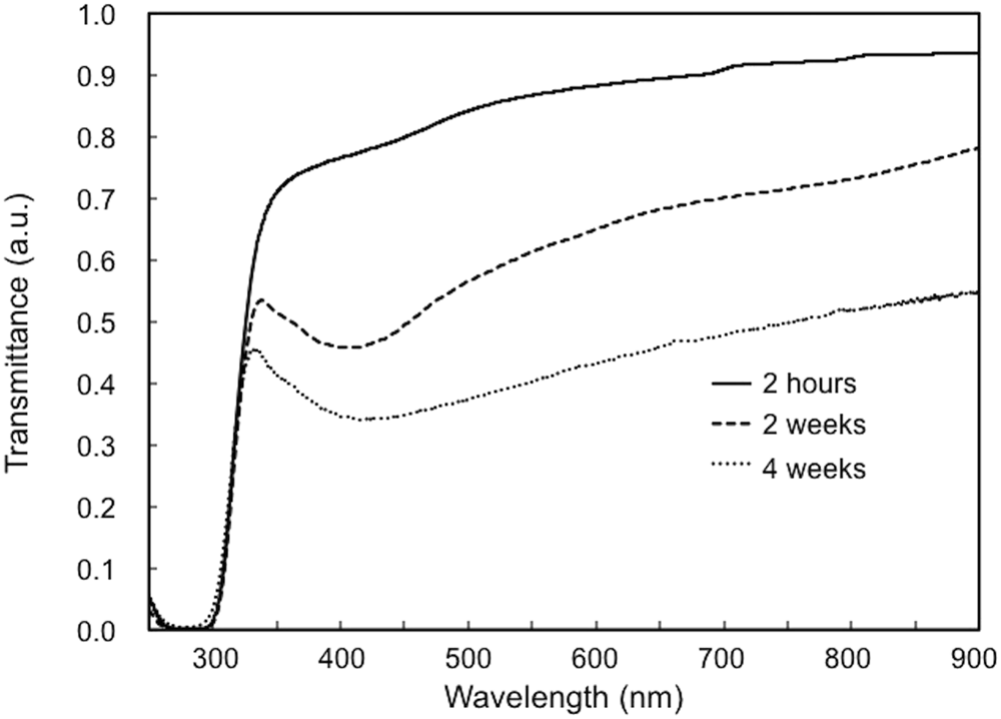
Transmission spectra of hydrogel containing silver ions, which was immersed in AgNO_3_ aqueous solution for 2 h (solid line), 2 weeks (dashed line), and 4 weeks (dotted line).

In the present study, although it is difficult to obtain the precise image due to the optical resolution limit of the microscope, the fabrication of the silver line structure was demonstrated with the scanning speed of 800 μm/s. Previously, Maruo *et al*.^14^ demonstrated the fabrication of silver microstructures by femtosecond laser-induced photoreduction in polyvinylpyrrolidone (PVP) films containing silver ions at laser scanning speeds of up to 200 μm/s. Lower scanning speeds of several micrometers to several tens of micrometers per second have also been used for the fabrication of structures by multi-photon photoreduction of metal ions by using a femtosecond laser^16–18,20–23,26–29^. In such previous studies, near-infrared wavelengths have been typically used for multi-photon photoreduction of metal ions. A limited number of papers have reported the use of visible wavelengths of femtosecond laser for the fabrication of metal microstructures^20,32,33^. Two-photon photoreduction, which is probably most dominant process in the fabrication, at high scanning speeds (as high as 800 μm/s) employed in the present study can be explained on the basis of the absorption property of the hydrogel. Figure 3 shows the transmission spectra of the hydrogel containing the silver ions. In our study, the silver line structures were fabricated after the hydrogel was immersed in a silver nitrate (AgNO_3_) aqueous solution for 2 h. The difference between linear and two-photon absorption is significantly large at 522-nm wavelength when compared with that at 800-nm wavelength, which results in an efficient two-photon photoreduction of metal ions at the focal point even at higher scanning speeds. In addition, a minimal value of the transmittance appeared at ~420 nm when the hydrogel was immersed in AgNO_3_ aqueous solution for longer periods (weeks), which suggests the formation of silver nanoseeds inside the bulk of the hydrogel. In previous experiments using near-infrared wavelengths, the photoreduction was enhanced by taking advantage of the multi-photon absorption of the silver nanoseeds prepared in advance^23,36^. In the present study, we were able to demonstrate the fabrication of silver microstructures without the silver nanoseeds by using a 522-nm-wavelength laser. These were efficiently fabricated in the local zone of the two-photon absorption.

### Optical properties of fabricated silver gratings inside a hydrogel

**Figure 4:**
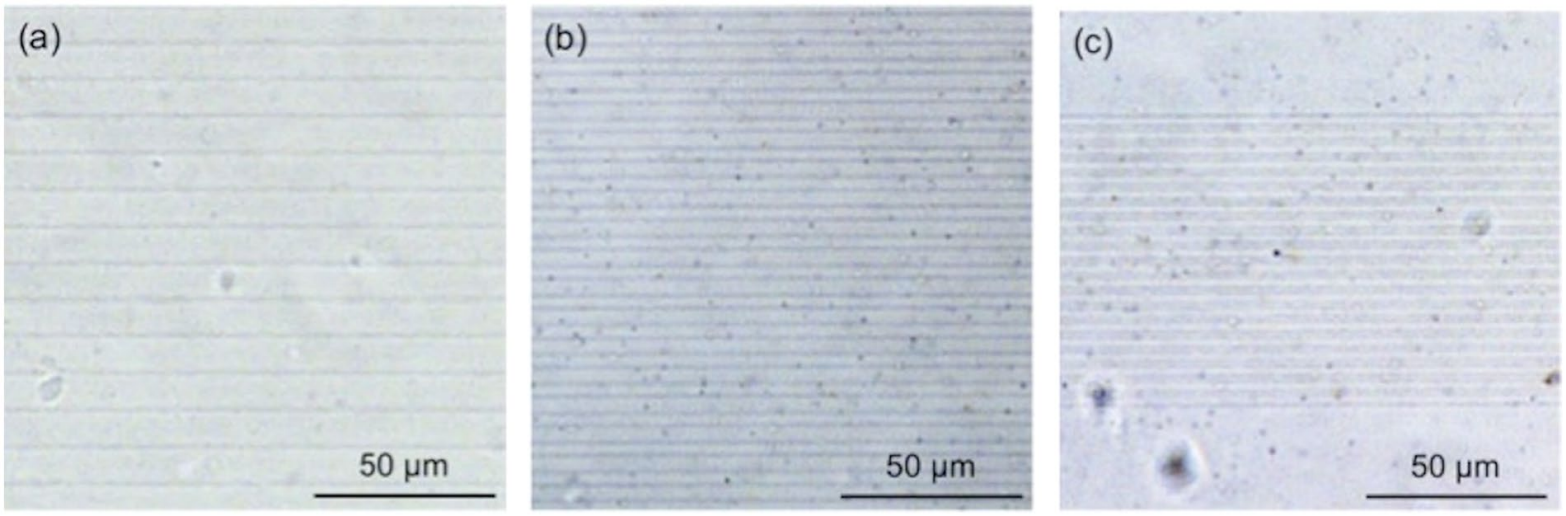
Bright-field microscope images of silver gratings fabricated inside the hydrogel. The spacing between adjacent lines in these patterns was (**a**) 10 μm, (**b**) 5 μm, and (**c**) 2 μm. The laser power and the scanning speed used for fabrication were 16.5 mW and 200 μm/s, respectively.

Figure 4 shows the bright-field microscope images of three silver gratings fabricated inside the hydrogels. The scanning speed and the laser power during the fabrication were 200 μm/s and 16.5 mW, respectively. The spacing between adjacent lines in these patterns is 10 μm (Fig. 4a), 5 μm (Fig. 4b), and 2 μm (Fig. 4c). The lines were found to be homogenous in all three gratings; however, it was difficult to discriminate several adjacent lines in the case of the grating period of 2 μm (Fig. 4c). This could be either due to the limitation in the optical resolution of the microscope or due to difficulty in forming isolated line structures because of the relative sizes of the focal spot diameter and the grating period. Additionally, the effect caused by the absorption cross section of the silver line structure needs to be considered, as the sizes of the line structures are in sub-micrometers^37^.

**Figure 5:**
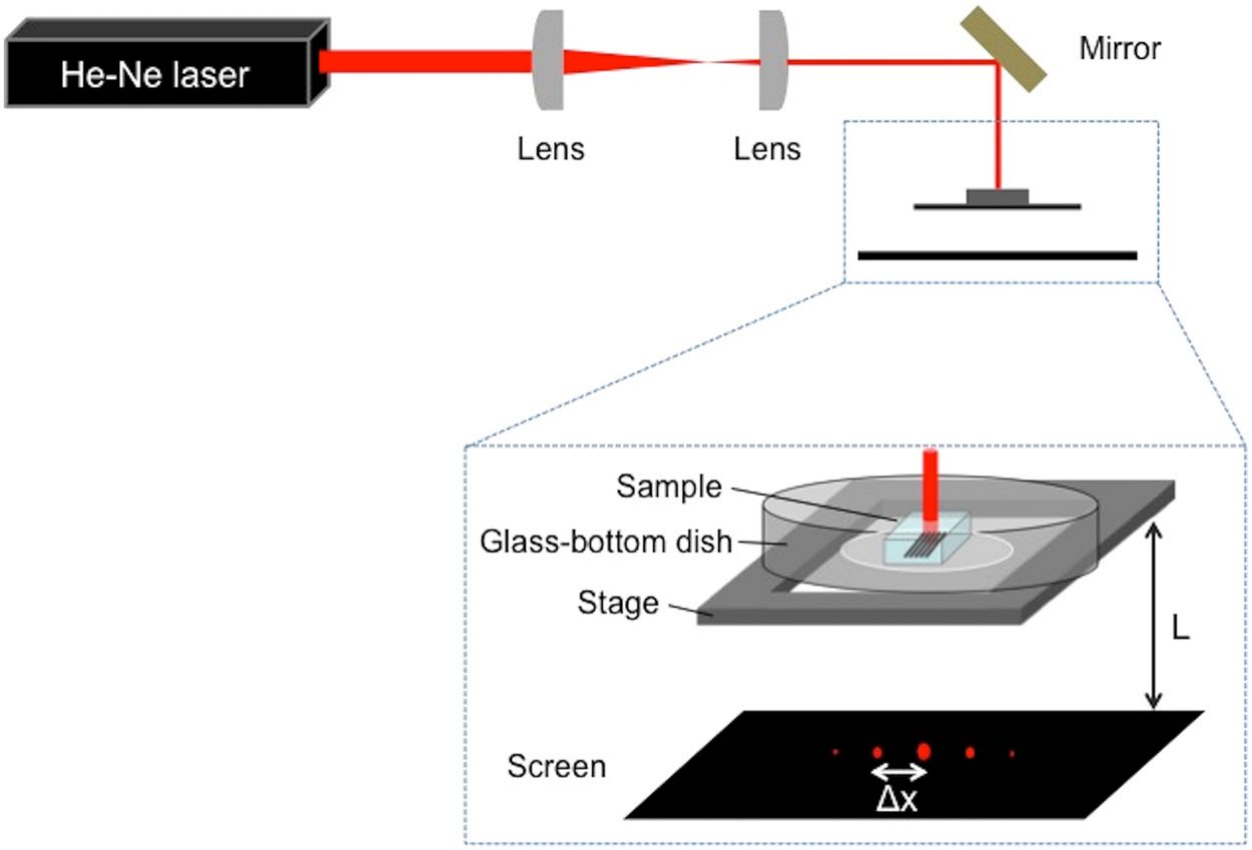
Schematic illustration of the experimental setup for evaluation of the optical properties of the fabricated structures. *L* and *Δx* denote the distance between the mounting stage and the screen and that between the diffraction spots, respectively.

**Figure 6:**
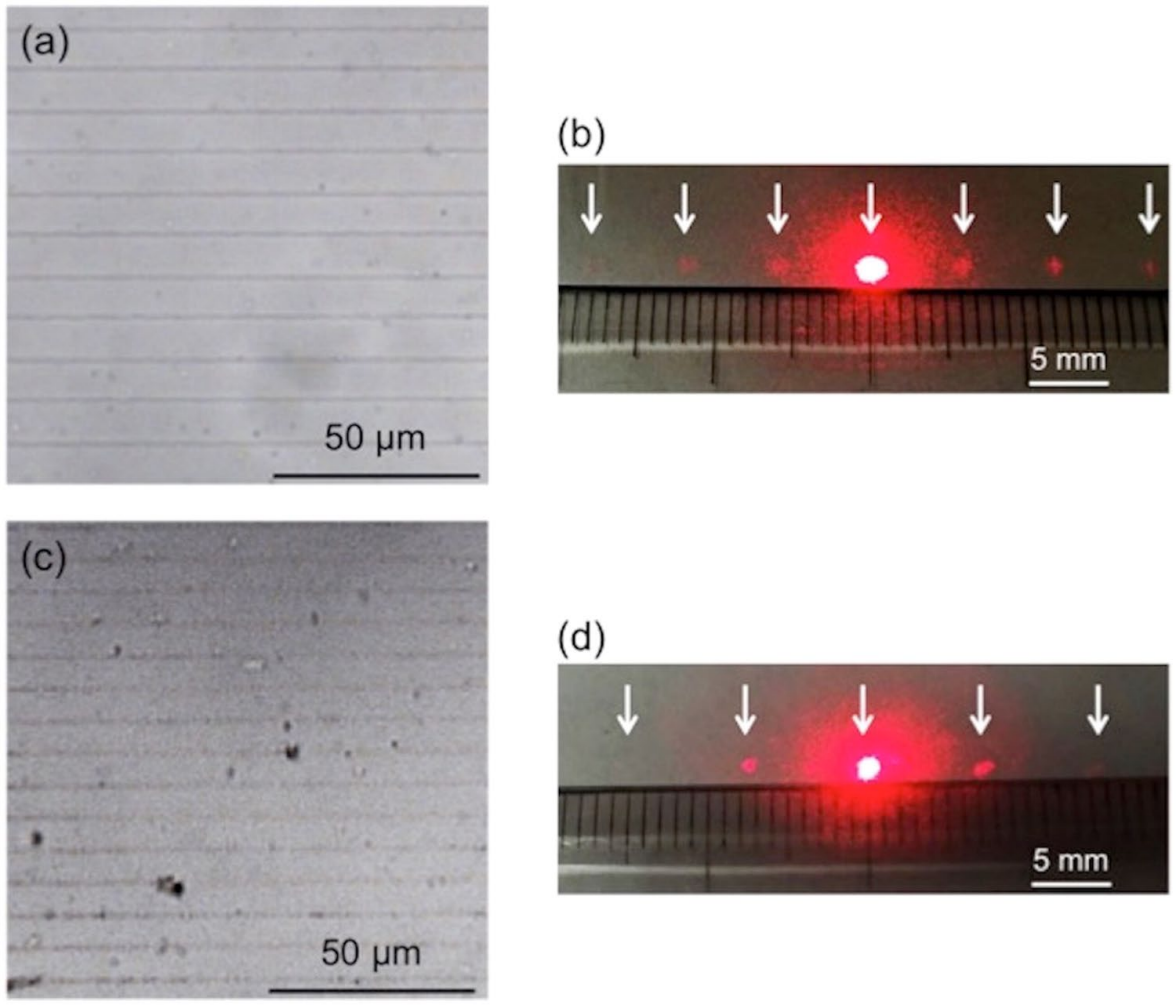
Bright-field microscope images of silver gratings inside the hydrogel and the corresponding optical diffraction patterns obtained before and after shrinking of the hydrogel. The laser power and the scanning speed used for fabrication were 16.5 mW and 200 μm/s, respectively. (**a**) Bright-field microscope image of silver grating fabricated inside the hydrogel before shrinking. Spacing between the adjacent lines was 10 μm. (**b**) Optical diffraction pattern obtained with the silver grating shown in (**a**). Distance between the diffraction spots was ~6.2 mm. (**c**) Bright-field microscope image of silver grating after shrinking of the hydrogel. Spacing between the adjacent lines decreased to 8 μm from the original value of 10 μm. (**d**) Optical diffraction pattern obtained with the silver grating shown in (**c**). Distance between the diffraction spots was ~7.5 mm. White arrows in (**b**) and (**d**) indicate the diffraction spots.

To evaluate the optical properties of the fabricated grating, we obtained the optical diffraction patterns using a CW laser. The schematic experimental arrangement is illustrated in Fig. 5. After fabrication of the silver grating, the hydrogel together with the grating was immersed in pure water and then placed on a glass-bottom dish immediately before optical evaluation. The laser beam (He-Ne) of wavelength 633 nm was incident on the top side of the grating fabricated within the hydrogel. The diffraction pattern of the light was projected on a screen placed below the hydrogel-containing dish. Figure 6a and b represent the bright-field microscope image of silver grating (10-μm period) fabricated within the hydrogel and its diffraction pattern obtained on the screen, respectively. The white arrows in Fig. 6b indicate the spots of the diffraction pattern. The spots were found to be equally spaced and the distance between the spots was measured to be ~6.2 mm. The diffraction spots were observed up to the 3rd order. The formation of equidistant spots validates the periodicity of the silver grating with a uniform silver line width. From applying the diffraction equation^38^ to the paraxial regime, the distance between the diffraction spots *Δx* was theoretically estimated to be 6.3 mm using the equation given below:

1 $${\rm{\Delta }}x=\frac{L\lambda }{d}$$

where *L* is the distance between the fabricated grating and the screen, *λ* is the wavelength of the incident laser light in air, and *d* is the grating period. The difference between the experimental and theoretical values was relatively small (~2%).

One of the advantages of using a hydrogel is that it changes in size depending on its water content. We also demonstrate the tunability of the fabricated silver grating by reducing the hydrogel’s water content. The hydrogel with the grating inside it was exposed to air in order to reduce its water content and, afterwards, the optical image and the diffraction pattern were obtained. Figure 6c and d show the bright-field microscope image of the silver grating within the hydrogel (post-shrinking) and the corresponding diffraction pattern, respectively. The grating period showed a decrease from 10 μm (original) to 8 μm (post-shrinking) while the straight-line shape of the structures was preserved; this is clearly evident in Fig. 6c. The size of the hydrogel shrank to ~80% of the original size. The value of *Δx* increased from 6.2 mm to 7.5 mm after shrinking of the hydrogel (Fig. 6d). From equation (1), the theoretical value of *Δx* was calculated to be 7.9 mm in the case of 8-μm grating period. Here, the difference in the experimental and theoretical values was estimated to be slightly higher (~5%) than the pre-shrunk value. It may be recalled that prior to the diffraction experiment, pure water was poured on the hydrogel in the glass-bottom dish immediately before the CW laser irradiation to prevent light scattering from its surface. Despite its brief contact for approximately few seconds, water is likely to induce a slight swelling of the hydrogel and consequently, a slight increase in the grating period. The values of the grating period and *Δx* returned to their original values of 10 μm and 6.2 mm, respectively (as in Fig. 6a and b), 15 min after pouring the water. From these results, the reversible nature of the characteristics of the fabricated silver grating and the responsive optical property of hydrogel with water content were confirmed. Further study is needed, but it is highly possible that the fabricated silver line structures were composed of nanoparticle aggregates as similar to other studies^17,18^.

The shrinkage of hydrogel depends not only on the water content, but also on the external environmental factors such as pH^7^, concentration^8^, and temperature^39^ of the solution in which it is immersed. While the results presented in this study pertain only to the water content, laser-based fabrication method using hydrogels can be potentially used to fabricate other sensing devices for measurements of pH, temperature, mechanical force/deformation, etc. Further, from the perspective of micro- and nanofabrication, the preciseness and the density of laser-based fabricated structures could be enhanced by shrinking the hydrogel after laser direct writing through processes that are likely to realize sizes beyond the theoretical diffraction limit of optical fabrication.

## Conclusion

We have demonstrated here an efficient method for fabricating a silver grating within poly(ethylene glycol) diacrylate (PEGDA) hydrogel using a 522-nm femtosecond laser for multi-photon photoreduction of silver ions. Silver line structures were fabricated at laser scanning speeds ranging from 10 to 800 μm/s. The silver grating fabricated inside the hydrogel produced an optical diffraction pattern of the incident CW laser beam with equally spaced diffraction spots; this indicated that a regular, periodic silver grating having uniform widths of silver line structures could be fabricated successfully. When the grating period decreased with the shrinkage of the hydrogel due to decrease in water content, the straight shapes of the line structures was preserved. The distance between the spots in the optical diffraction pattern changed corresponding to the shrinkage, which indicated the tunable optical property of the fabricated structure. Our study shows that the femtosecond laser-based photoreduction technique has the potential to realize tunable and highly precise optical devices by taking advantage of the hydrogel characteristics.

## Methods

### Material preparation

PEGDA (average molecular weight 6000) and the photoinitiator Irgacure 2959 were purchased from Sigma-Aldrich Co. LLC (St. Louis, MO). PEGDA of weight 0.1 g was dissolved in 1 ml of pure water containing 1% photoinitiator and stirred for 15 min. The solution placed in a mold was illuminated by a 365-nm light from a UV lamp for 20 min to induce photo-cross-linking. After the hydrogel was immersed in pure water for 2 h, it was immersed in AgNO_3_ aqueous solution (40 mg/ml) for 2 h to allow silver ions to permeate inside the hydrogel. The hydrogel of 5 mm in length, 5 mm in width, and 3 mm in thickness was used for the experiments of the laser parameter dependence on the fabrication. For experiments of the optical properties, the hydrogel of 5 mm in length, 5 mm in width, and 2 mm in thickness was used.

### Fabrication of silver line structures by femtosecond laser direct writing

A femtosecond laser (HighQ-2-SHG, Spectra-Physics, Inc., Santa Clara, California) operating at 522-nm wavelength, 63-MHz repetition rate, and 192-fs pulse duration was used for the fabrication of silver line structures by multi-photon photoreduction of silver ions. The schematic illustration of the experimental setup is shown in Fig. 1. The hydrogel containing silver ions was placed in a glass-bottom dish after briefly rinsing it with pure water. Femtosecond laser pulses were focused inside the hydrogel by using a water immersion objective lens (numerical aperture (NA): 1.0, working distance: 2.0 mm; Olympus). Linear structures were fabricated using a computer-controlled, three-axis encoded (XYZ) motorized stage. A real-time observation system with a CMOS camera was used to adjust the position of the focal point.

### Characterization

Silver line structures fabricated inside the hydrogel were observed by a bright-field microscope (Eclipse Ti-E, Nikon, Tokyo, Japan). 10× (NA: 0.45) and 60× (NA: 0.95) magnification microscope objectives were used for obtaining the images. The line width of the fabricated line structure was defined by the FWHM obtained from the gray values of the bright-field microscope images.

### Measurement of transmittance

Transmission spectrum of the hydrogel containing silver ions was measured using ultraviolet-visible-near infrared spectrophotometer (UV-3600 plus, Shimadzu, Kyoto, Japan) for wavelengths ranging from 250 nm to 900 nm.

### Optical property evaluation

Figure 5 shows the setup for the evaluation of the optical property of the fabricated structures. The hydrogel in which the silver grating was fabricated was placed in a glass-bottom dish. Pure water was added to the dish to prevent light scattering from the surface of the hydrogel. Laser beam from a He-Ne laser (633 nm) was directed on the top side of the grating contained in the hydrogel. The laser power and beam diameter of the He-Ne laser was 2 mW and 1 mm, respectively. The light diffraction pattern from the silver grating was projected on a screen placed 100 mm below the hydrogel.

### Data Availability

All data generated or analysed during this study are included in this published article.
